# An Exceptional Case of Light Chain Only Variant of Proliferative Glomerulonephritis with Monoclonal Immunoglobulin Deposits Secondary to Chronic Lymphocytic Leukemia: A Case Report and Review of the Literature

**DOI:** 10.1155/2022/9207282

**Published:** 2022-10-19

**Authors:** José C. De La Flor, Jacqueline Apaza, Francisco Díaz, Edna Sandoval, Tania Linares, Alexander Marschall, Patricia Núñez, Andrea Cecilia Rivas-Nieto, Elisa Ruiz

**Affiliations:** ^1^Department of Nephrology, Hospital Central Defense Gomez Ulla, Madrid, Spain; ^2^Department of Nephrology, Hospital Fuenlabrada, Madrid, Spain; ^3^Department of Anatomic Pathology, Hospital Gregorio Marañon, Madrid, Spain; ^4^Department of Hematology, Hospital Central Defense Gomez Ulla, Madrid, Spain; ^5^Department of Cardiology, Hospital Central Defense Gomez Ulla, Madrid, Spain; ^6^Department of Nephrology, Hospital Cayetano Heredia, Lima, Peru

## Abstract

We present the case of an 86-year-old Caucasian male with an 11-year history of low-grade chronic lymphocytic leukemia (CLL) presenting with nephrotic syndrome (NS). Renal biopsy findings showed a diffuse mesangial and endocapillary proliferative glomerulonephritis (GN) lesion with fine granular deposits, consistent with a rare morphologic variant of proliferative glomerulonephritis with monoclonal immunoglobulin deposits (PGNMID)-lambda light chain (LC) only. Monthly combination therapy of rituximab (500 mg/m^2^ on day 1), fludarabine (30 mg/m^2^ on days 1–3), and cyclophosphamide (750 mg/m^2^ on days 1–3) was administered. Five courses of this regimen resulted in hematological remission, as well as a partial renal response with a reduction in the spot urine protein-to-creatinine ratio (UPCR) of 815.3 mg/g (reduction > 50% proteinuria without improvement in kidney function). This condition is a rare morphological variant of PGNMID, poorly described in CLL patients. We review the literature and suggest that this case provides sheds light on the unknown pathophysiological mechanisms of monoclonal immunoglobulins (MIg)-mediated glomerular damage in CLL patients, and may be helpful for the investigation of a more effective treatment.

## 1. Introduction

Chronic lymphocytic leukemia (CLL) is one of the most common chronic lymphoproliferative disorders, accounting for 30% of all leukemias, with an incidence of 4–5 cases per 100,000 habitants per year, and an average age at the time of diagnosis of 60 years [1], making it the most common leukemia in the elderly. CLL is a disease of neoplastic clonal B cells, primarily involving bone marrow, peripheral blood, lymph nodes, liver, and spleen. Extranodal manifestations of CLL are rare and renal involvement is relatively uncommon. The spectrum of kidney pathology includes immune-mediated glomerulonephritis (GN), interstitial infiltration, tubular obstruction, and drug toxicity.

The association between glomerular disease and CLL is apparently rare despite the relative frequency of this type of leukemia [2]. In CLL, the proliferation of the *B*-cell clone does not lead to plasma-cell terminal differentiation. The incidence of GN in CLL is low and the physiopathology links with the hematologic malignancy appear most often hypothetical [3]. The most frequently reported glomerular lesions are membranoproliferative glomerulonephritis (MPGN) and membranous nephropathy. Less commonly, minimal change disease (MCD), focal segmental glomerulosclerosis, amyloidosis, cryoglobulinemic glomerulonephritis, and immunotactoid/microtubular glomerulopathies, have been described [4]. Cases where the monoclonal immunoglobulins (MIg) secreted by the clone of *B* cells may be directly and indirectly involved in the pathogenesis of the glomerular lesions, would be categorized as monoclonal gammopathy lesions of renal significance (MGRS), according to the recent classification [5]. The lesions are governed by the physicochemical properties of the pathogenic MIg rather than the proliferation rate or tumor burden of the *B*-lymphocyte that produces them. MGRS-related kidney lesions are classified according to the characteristics of the MIg deposits on electron microscopy as follows: organized, nonorganized, or absent deposits. The proliferative glomerulonephritis with monoclonal immunoglobulin deposits (PGNMID) is a form of MGRS-associated lesion showing nonorganized MIg granular electron-dense deposits in absence of cryoglobulins [6]. This entity, described by Nasr et al. [7], was characterized by diffuse proliferative (mesangial and endocapillary), membranoproliferative or membranous features with fine granular deposits, mimicking immune complex-type deposits on electron microscopy. In most PGNMID cases, the pathogenic deposited MIg is IgG, but in rare cases, it can be IgA or IgM [8]. However, cases of PGNMID with deposition of nonorganized MIg light chains (LC) only (PGNMID-LC), particularly associated with pathogenic plasma cell clones, are exceptional [9]. Herein, we describe a rare case of CLL with a *B*-cell clonal disorder showing PGNMID-lambda light chain on renal biopsy.

## 2. Case Report

Our patient is an 86-year-old Caucasian male with an 11-year history of low-grade CLL being followed conservatively by a hematologist. Over 3 months, he developed progressive edema in his lower legs, asthenia, fatigue, and uncontrolled arterial hypertension (AHT). His medication included angiotensin II receptor blockers (ARBs), furosemide, and statin.

On admission, physical examination revealed a blood pressure of 180/87 mmHg, a respiratory rate of 24 breaths/min, oxygen saturation of 96% while breathing ambient air, pulmonary rales, and peripheral edema. His laboratory values indicated a creatinine (Cr) level of 1.67 mg/dl, estimated GFR (eGFR) 36.5 mL/min/1.73 m^2^ by chronic kidney disease epidemiology (CKD-EPI) formula, and hypoalbuminemia without hypercholesterolemia. His urine showed 15–25 red blood cells per high-power field (RBC/HPF) with dysmorphic cells. The 24-hour urine protein excretion was 7.72 g with a significant disproportion of spot urine protein-to-creatinine ratio (UPCR) and urine albumin-to-creatinine ratio (UACR) of 7614.94 and 2635.39 mg/g, respectively. Serum protein immunofixation/electrophoresis (IFE) revealed a trace band positive for IgG-kappa and IgG-lambda *M*-protein. Urine IFE also showed only a weak band of IgG-lambda. Other laboratory findings revealed the following: leukocyte count, 6.92 × 10^3^/uL with 35.4% lymphocyte; erythrocyte count, 3.56 × 10^6^/uL with his other hematologic cell lines being normal. The rest of his investigations were normal (cryoglobulins, hepatitis B and C, antinuclear antibody (ANA), antineutrophil cytoplasmic antibody (ANCA), rheumatoid factor, serum complement-3 (C3), and complement-4 (C4). The patient's creatinine rose to 2.3 mg/dl during the in-hospital stay after starting depletive treatment, probably associated with hemodynamic changes due to antiproteinuric medication. Other laboratory test results are shown in Table 1.

**Figure 1:**
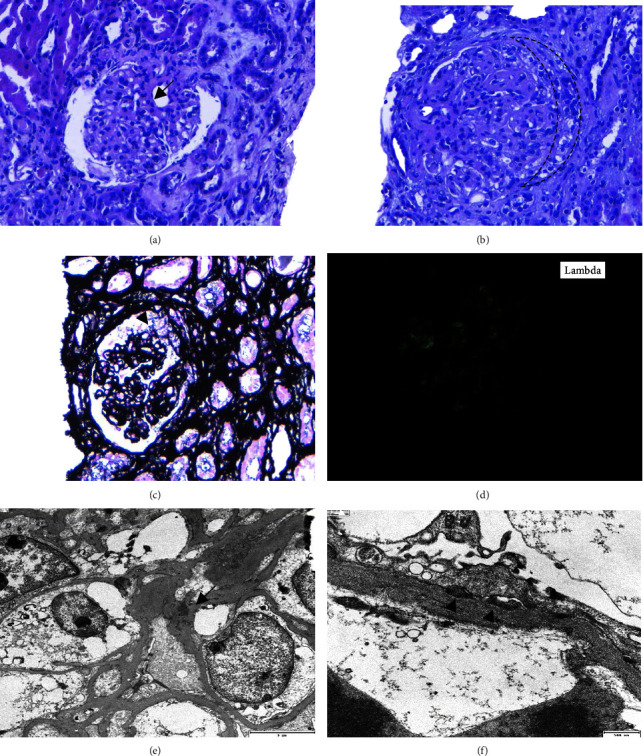
Light microscopy shows a global increase of mesangial matrix, with focally mesangial hypercellularity (arrow). (a) Hyalinosis of the afferent arteriole was observed (asterisk) (*H*-*E*, 200*x*). (b) The glomerulus shows irregular capillary wall thickening and a cellular crescent (dashed crescent) (*H*-*E*, 200*x*). (c) Methanamine-silver stain (200x) shows a glomerulus with a cellular crescent (arrowhead). (d) Direct immunofluorescence showing bright granular staining of glomerular mesangium for lambda light chain (lambda++/kappa+, 200*x*). (e) Electron microscopy revealed large electron-dense nonorganized deposits in mesangial areas (arrow) (10000*x*). (f) Electron microscopy showing segmental subendothelial mesangial deposits (arrow-head) (10000*x*).

A renal biopsy was performed and 38 glomeruli were examined under light microscopy (LM), 17 of which (44%) were globally sclerotic. All of the remaining glomeruli exhibited a global and diffuse expansion of the mesangial matrix, with a focally (<50% of glomeruli) increase of mesangial cells. A variable degree of pericapsular fibrosis was observed. The capillary loops were slightly thickened and no double contours, endocapillary proliferation, or intravascular thrombi were observed. In addition, three glomeruli showed extracapillary epithelial cell proliferation, two of them cell lesions and one fibrocellular. A mild degree of interstitial scarring (30%) and tubular atrophy were also noted. A mild infiltration of lymphocytes, histologically nonspecific, occupying less than 5% of the cortical surface was observed. Mild arteriolosclerosis was seen. No intravascular thrombi or vasculitis was observed. Congo red and thioflavin staining were negative (Figures 1(a)–1(c)). Marked intensity for the lambda light chain (2+) and weak intensity for the kappa light chain (1+) in both the mesangium and peripheral capillary loops were demonstrated by frozen tissue immunofluorescence (IF-F). In contrast, the IF was negative for IgG, IgM, IgA, C1q, and C3. On the other hand, the IF on pronase-digested paraffin-embedded (IF-P) sections showed no subendothelial or mesangial deposits of IgG, IgM, or IgA (Figure 1(d)). Electron microscopy (EM) revealed large electron-dense nonorganized deposits in mesangial and subendothelial areas (Figures 1(e) and 1(f)). The immunohistochemistry technique for DNAJB9 was performed with a negative result.

The bone marrow (BM) aspiration showed increased cellularity. The immunophenotypic study showed that 45.4% of the total cellularity was lymphocytes. Of this population, 27.7% were mature lymphocytes of monoclonal origin with moderate intensity lambda light chain restriction, with an immunophenotype compatible with CLL (CD19+, CD45+, CD5++, CD200+++, and weakly positive for CD20). The few plasma cells revealed a phenotype without abnormalities or restriction of intracytoplasmic light chains. A fluorescence in situ hybridization (FISH) cytogenetic study was performed in bone marrow, being negative for BCL-2 and *t* (11; 14). A positron emission tomography-computed tomography scan (PET/CT) did not suggest lymphoproliferative syndrome, solitary plasmacytoma, or other extramedullary affectations.

Therefore, the present case was diagnosed as lesions PGNMID-Lambda LC only associated with CLL. Monthly combination therapy of rituximab (500 mg/m^2^ on day 1), fludarabine (30 mg/m^2^ on days 1–3), and cyclophosphamide (750 mg/m^2^ on days 1–3) was administered. Five courses of this regimen resulted in hematological remission, as well as a partial renal response with a reduction in the UPCR of 815.3 mg/g (reduction >50% proteinuria without improvement in kidney function).

## 3. Discussion

In this report, we describe a case of CLL presenting with NS. Renal biopsy findings showed a diffuse mesangial and endocapillary proliferative GN lesion with fine granular deposits, consistent with a rare morphologic variant of PGNMID-Lambda LC only. The LM, IF and EM findings of our patient described MGRS-related renal lesions with deposit Ig nonorganized. These findings were completely different from those of monoclonal immunoglobulin deposition disease (MIDD). MIDD comprises a group of diseases characterized by the deposition of light chains (LCDD), heavy chains (HCDD), or both light and heavy chains deposits (LHCDD) are seen along both the glomerular basement membrane (GBM) and the tubular basement membrane (TBM). In addition, LCDD is characterized by nodular mesangial glomerulosclerosis resembling Kimmelstiel–Wilson nodules and the continuous deposition of fine, electron-dense granular materials along the inner aspect of the glomerulus [10].

The incidence of NS in patients with CLL is approximately 1% to 2% [11]. While the renal complications of plasma cell dyscrasia have been well described, most information on patients with CLL is derived from case reports. There are two large case series published in the literature [2, 4]. The larger of the two was that of Strati et al. [4], who completed a retrospective analysis of patients with CLL or monoclonal *B*-cell lymphocytosis that underwent kidney biopsy for renal insufficiency and/or NS. Between January 1995 and June 2014, 49 of 4,024 (1.2%) patients with CLL (*n* = 44) or monoclonal *B*-cell lymphocytosis (*n* = 5) had a renal biopsy: 34 (69%) for renal insufficiency and 15 (31%) for nephrotic syndrome. The most common findings were MPGN (*n* = 10, 20%), chronic lymphocytic leukemia interstitial infiltration as primary etiology (*n* = 6, 12%), thrombotic microangiopathy (*n* = 6, 12%), and minimal change disease (*n* = 5, 10%). MPGN is one of the most frequent kidney diseases described in association with CLL, which is caused by cryoglobulinemia, predominantly type II [12], or may be associated with deposition of a MIg secreted by leukemia *B* cells [3, 11], recently included in a new diagnostic term denominated MGRS [6]. The MGRS is pathogenically characterized by the proliferation of B-lymphocyte clones or plasma cell clones that synthesize and secrete a MIg or its components (light or heavy chains), with the ability to cause damage at the tubular, glomerular, vascular, and interstitial compartments through direct (deposition) or indirect (alterations of the alternative complement pathway) mechanisms, constituting a heterogeneous group of entities [13, 14].

In 2004, Nasr et al. [15] described for the first time a new type of glomerular injury related to MIg deposition without involving TBM, which they classified as PGNMID. PGNMID are characterized by deposits typically composed of intact MIg with both Ig heavy and light chains, particularly by IgG (PGNMID-IgG) or exceptionally by deposits of nonorganized MIg light chains (LC) only (PGNMID-LC). According to Nasr et al. [9] in a series of 17 cases with PGNMID-LC, based on the pathologic findings by LM and EM, they described several notable pathological and immune-hematologic differences between PGNMID-LC and the considerably more common variant-PGNMID-IgG primarily, the deposits on IF in PGNMID-LC stain only for Ig-LC without staining for IgG neither other Ig type (IgA or IgM). These observations are consistent with our findings, as we confirmed the absence of IgG, IgA, or IgM by IF-P and EM. Secondly, both in PGNMID-IgG and PGNMID-LC, the codeposit of C3 is nearly always described, while the deposits of C1q are more frequent in PGNMID-IgG than PGNMID-LC [9]. In contrast, the present case did not show C3 or C1q codeposits on IF or IF-P. At this point, the liquid chromatography-mass spectrometry (LC-MS) study would have been very useful. Leung et al., in the expert consensus document of the International Kidney and Monoclonal Gammopathy Research Group, recommend the usefulness of LC-MS for the diagnosis of MGSR lesions when IF studies have negative findings [5], as in our case. However, this technique is not available in our center. Thirdly, the subendothelial deposits in most cases of PGNMID-IgG are small and best seen by EM, while that in PGNMID-LC are frequently large and easily identified by LM [9], these findings are in line with the biopsy of our case, except for the presence of diffuse proliferative mesangial and endocapillary lesions. Nasr et al. [9] hypothesized that this could be due to the higher concentration of circulating MIg levels in PGNMID-LC than in PGNMID-IgG and this may also reflect differences in the mechanisms of complement activation induced by the deposition of monoclonal LCs. Lastly, clonal identification is most difficult in patients who have PGNMID-IgG, in more than 80%, pathological clones of B cells and plasma cells are not detected in BM at diagnosis or on follow-up [8, 9, 15]. Nasr et al. [9] reported that the underlying clone in all PGNMID-LC cases was a plasma cell clone, these nephropathic clone was detected in the BM in 88% (14 of 16), coinciding with other case reports [16–20]. Unlike in our case, proliferative glomerulonephritis was caused by the secretion and deposition of lambda LC in a patient with CLL, in which the pathological clone was a *B*-lymphocyte. Therefore, to the best of our knowledge, this case could be one of the first to describe PGNMID-LC associated with CLL.

Regarding the hematological characteristics of PGNMID-LC, the serum free light chain (FLC) ratio was abnormally elevated in almost 83% (10 of 12 patients tested) of PGNMID-LC cases reported by Nasr et al. [9], including 3 patients with a serum FLC ratio > 100, and normal in 2 (17%) patients. On the other hand, in a study by Buthani et al. [8], only 20% of patients with PGNMID-IgG presented abnormal FLC. In our patient, we found two serum bands of monoclonal IgG-kappa and IgG-lambda, urine IFE with a faint band of IgG-lambda, and renal deposits of lambda LCs in mesangial and capillary loops. In addition, the serum FLC kappa and lambda were slightly elevated with a normal serum FLC ratio. On the other hand, our patient did not present hypocomplementemia or alterations in the study of the alternative pathway of complement (APC). In the cohort of PGNMID-LC reported by Nasr et al. [9], they detected hypocomplementemia (low C3 with normal C4) in 6 (50%) of 12 patients. Four of these patients had complement factor B (CFB), factor H (CFH), and factor I (CFI) levels within normal limits and none had detectable C3 nephritic factor (C3FN) or antifactor H IgG autoantibody.

The pathogenesis of PGNMID-LC remains uncertain. Nasr et al. [9], suggest that PGNMID-LC is mediated by local APC activation in the glomeruli by the MIg-LC, which then activates downstream inflammatory mediators that promote glomerular proliferation, leukocyte infiltration, and glomerular basement membrane damage and reparative changes leading to MPGN. However, in our case, there is relevant data, such as the absence of hypocomplementemia and MPGN lesions associated with a normal serum FLC ratio, which leads us to think that there may be other mechanisms of glomerular damage mediated by MIg not yet known in this rare variant of PGNMID-LC.

As in other forms of MGRS-related lesions, the treatment strategies are based on chemotherapy that must be adapted to the nature of the cell clone, both lymphocytic or plasma, renal function, and the presence or absence of extrarenal involvement [6]. CLL is a malignancy characterized by clonal proliferation of B-lymphocytes that are often CD20 positive [21]. Therefore, the abnormal lambda LC produced by the malignant clone is responsible for the LC deposition and subsequent proliferation observed in our case with PGNMID-LC. Rituximab (a monoclonal antibody to CD20) produces depletion of B-lymphocytes in patients with CLL [21] and would be expected to reduce the production and deposition of pathogenic MIg-LC. Most previously reported cases only refer to the management of rituximab in patients with CLL and PGNMID-IgG [22, 23]. However, there are no cases describing CLL and PGNMID-LC. Two of the cases of CLL and PGNMID-IgG show ambiguous results. Barbour et al. [22] reported two cases treated with rituximab with reduced proteinuria and improved renal function, in addition to a complete hematologic response. On the other hand, Bhat et al. [23] described 3 patients, 2 of them experienced partial remission of their disease and 1 patient was able to come off hemodialysis therapy after treatment with 7 and 11 biweekly doses of rituximab, 375 mg/m. Both patients subsequently experienced a relapse of their hematologic and renal diseases and eventually progressed to end-stage renal disease and death. A third patient was treated with two 1,000 mg infusions of rituximab separated by 2 weeks, with sustained partial remission at 18 months follow-up. In a small, multicenter, retrospective study of 17 patients with CLL and PGNMID-LC by Nasr et al. [9], plasma cell-targeted chemotherapy (mostly bortezomib-based therapy) resulted in complete or partial renal recovery in 6 of the 10 patients treated, whereas none of 5 patients given nontargeted treatment recovered kidney function. As in other forms of MGRS-related kidney lesions, renal response depended on hematologic response [6]. Our patient was treated with *B*-lymphocyte cell-targeted chemotherapy (mostly Rituximab-based therapy) and experienced partial renal remission and complete hematologic response.

In conclusion, we describe an exceptional case of PGNMID-Lambda LC with CLL. This condition is a rare morphological variant of PGNMID, poorly described in CLL patients. This case provides sheds light on the unknown pathophysiological mechanisms of MIg-mediated glomerular damage in CLL patients and may be helpful for the investigation of a more effective treatment.

## Figures and Tables

**Table 1:** Laboratory findings on admission.

		Reference range/Unit
WBC	6920	U/L
Hemoglobin (Hb)	11	12–16 g/dL
Platelet count (PLt)	131	10^3^/uL
Reticulocytes count	2.77	2–4%
Erythrocyte count	3.56	4.2–5.8 10^6^/uL
Lactate dehydrogenase (LDH)	180	135–214 IU/L
Coombs test	Negative	NA
Total bilirubin	0.5	0.1–1 mg/dL
Total protein (TP)	5.5	6.4–8.7 g/dL
Serum albumin (Alb)	2.56	3–5.5 g/dL
GOT	21	5–32 IU/L
GPT	12	5–33 IU/L
Urea	98	17–60 mg/dL
Creatinine	1.67	0.6–1.2 mg/dL
Na	138	135–145 mmol/L
K	5.2	3.5–5.5 mmol/L
Cl	99	95–110 mmol/L
CRP	1.5	0.1–0.5 mg/dL
Hbs-Ag	Negative	NA
HCV-Ab	Negative	NA
HIV	Negative	NA
CH50	50	40–90 U/ml
CFH	250	225–760 *μ*g/mL
Autoantibodies CFH	Negative	<18 AU/L
C3 nephritic factor (C3NF)	Negative	Ratio >1.022
C3	116	90–180 mg/dL
C4	22.5	10–40 mg/dL
RF	Negative	<15 IU/ml
ANA, anti-dsDNA, ANCA, and cryoglobulin	Negative	NA
Anti-GBM	Negative	<1 AI
Anti-PLA2R Ab (ELISA)	Negative	NA
Beta 2 microglobulin	1.09	<0–20 mg/dL
IgG	1200	800–1600 mg/dL
IgA	151	70–400 mg/dL
IgM	67.7	90–180 mg/dL
UPCR	7614.94	<20 mg/g
UACR	2635.39	<30 mg/g
Urine red blood cells	15–25	/HPF
24 h urine total protein excretion	7.72	<0.15 g/24 h
SPEP M-protein concentration	2 M-protein: 1.5 and 2.9%	NA
SIF	IgG *κ*, IgG *λ*	NA
Urine immunofixation electrophoresis	IgG *λ*	NA mg/dL
Urine IgG	52.70	NA mg/dL
FLC *κ*	30.1	4.90–13.70 mg/L
FLC *λ*	53	7.60–19.50 mg/L
FLC *κ*/*λ*	0.57	0.27–1.67

AI: activity index; AU: arbitrary units; NA: not applicable; WBC: white blood cells; GOT: glutamate-oxaloacetate transaminase; GPT: glutamate pyruvate transaminase; Na: sodium serum; K: potassium serum; Cl: chloride serum; CRP: C—reactive protein; CH50: complement hemolytic activity-50; CFH: complement factor H; C3NF: complement-3 nephritic factor; C3: complement-3; C4: complement-4; RF: rheumatoid factor; ANA: antinuclear antibody; anti-dsDNA: anti-double stranded DNA antibody; ANCA: anti-neutrophil cytoplasmic autoantibody; anti-GBM: anti-glomerular basement membrane; anti-PLA2R Ab: anti-phospholipase a2 receptor antibody; Ig: immunoglobulin; UPCR: spot urine protein-to-creatinine ratio; UACR: spot urine albumin-to-creatinine ratio; ELISA: enzyme-linked immunosorbent assay; SPEP: serum protein electrophoresis; SIF: serum immunofixation; FLC: free light chain; *κ*: kappa; *λ*: lambda; HPF: high-power field.

## Data Availability

The data used to support the findings of this study are included within the article.
